# Using Extended Genealogy to Estimate Components of Heritability for 23 Quantitative and Dichotomous Traits

**DOI:** 10.1371/journal.pgen.1003520

**Published:** 2013-05-30

**Authors:** Noah Zaitlen, Peter Kraft, Nick Patterson, Bogdan Pasaniuc, Gaurav Bhatia, Samuela Pollack, Alkes L. Price

**Affiliations:** 1 Department of Medicine, Lung Biology Center, University of California San Francisco, San Francisco, California, United States of America; 2 Department of Epidemiology, Harvard School of Public Health, Boston, Massachusetts, United States of America; 3 Department of Biostatistics, Harvard School of Public Health, Boston, Massachusetts, United States of America; 4 Broad Institute of Harvard and Massachusetts Institute of Technology, Cambridge, Massachusetts, United States of America; 5 Interdepartmental Program in Bioinformatics Pathology and Laboratory Medicine, University of California Los Angeles, Los Angeles, California, United States of America; The University of Queensland, Australia

## Abstract

Important knowledge about the determinants of complex human phenotypes can be obtained from the estimation of heritability, the fraction of phenotypic variation in a population that is determined by genetic factors. Here, we make use of extensive phenotype data in Iceland, long-range phased genotypes, and a population-wide genealogical database to examine the heritability of 11 quantitative and 12 dichotomous phenotypes in a sample of 38,167 individuals. Most previous estimates of heritability are derived from family-based approaches such as twin studies, which may be biased upwards by epistatic interactions or shared environment. Our estimates of heritability, based on both closely and distantly related pairs of individuals, are significantly lower than those from previous studies. We examine phenotypic correlations across a range of relationships, from siblings to first cousins, and find that the excess phenotypic correlation in these related individuals is predominantly due to shared environment as opposed to dominance or epistasis. We also develop a new method to jointly estimate narrow-sense heritability and the heritability explained by genotyped SNPs. Unlike existing methods, this approach permits the use of information from both closely and distantly related pairs of individuals, thereby reducing the variance of estimates of heritability explained by genotyped SNPs while preventing upward bias. Our results show that common SNPs explain a larger proportion of the heritability than previously thought, with SNPs present on Illumina 300K genotyping arrays explaining more than half of the heritability for the 23 phenotypes examined in this study. Much of the remaining heritability is likely to be due to rare alleles that are not captured by standard genotyping arrays.

## Author Summary

Phenotype is a function of a genome and its environment. Heritability is the fraction of variation in a phenotype determined by genetic factors in a population. Current methods to estimate heritability rely on the phenotypic correlations of closely related individuals and are potentially upwardly biased, due to the impact of epistasis and shared environment. We develop new methods to estimate heritability over both closely and distantly related individuals. By examining the phenotypic correlation among different types of related individuals such as siblings, half-siblings, and first cousins, we show that shared environment is the primary determinant of inflated estimates of heritability. For a large number of phenotypes, it is not known how much of the heritability is explained by SNPs included on current genotyping platforms. Existing methods to estimate this component of heritability are biased in the presence of related individuals. We develop a method that permits the inclusion of both closely and distantly related individuals when estimating heritability explained by genotyped SNPs and use it to make estimates for 23 medically relevant phenotypes. These estimates can be used to increase our understanding of the distribution and frequency of functionally relevant variants and thereby inform the design of future studies.

## Introduction

Although genome-wide association studies (GWAS) have resulted in the discovery of thousands of novel associations of loci to hundreds of phenotypes [1], concerns have been raised about the finding that these loci appear to explain a relatively small proportion of the estimated heritability, the fraction of phenotypic variation in a population that is due to genetic variation [2]. This has led to considerable speculation by researchers about the genetic basis of complex human phenotypes and the “missing heritability”, i.e. the fraction of heritability not accounted for by the associations discovered to date [3], [4], [5], [6], [7], [8], [9]. Among the proposed explanations for missing heritability is the existence of many presently unidentified common variants with small effect sizes, rare variants not captured by current genotyping platforms, structural variants, epistatic interactions, gene-environment interactions, parent-of-origin effects, or inflated heritability estimates [3], [5], [10]. Studies that examine the sources of missing heritability can help researchers to evaluate the prospects of future studies focusing on common versus rare variation and thereby devise effective strategies to discover the remaining sequence variants that affect disease risk and other aspects of phenotypic variation in humans.

The narrow-sense heritability of a phenotype (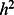) is the fraction of phenotypic variance that can be described by an additive model over the set of SNPs that are functionally related to the phenotype (i.e. the causal SNPs) [11]. It is commonly estimated by comparing the phenotypic correlation of monozygotic (MZ) to that of dizygotic (DZ) twins. The difference between 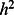 and the fraction of phenotypic variance accounted for by variants discovered by means of GWAS (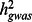) is the so-called missing heritability. Recently, Yang et al [12] developed a method to estimate the variance explained by all SNPs on a genotyping platform including those that are not genome-wide significant (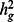), representing the limit of 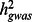 for infinite sample size.

There are two major challenges in comparing 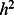 and 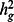 to quantify missing heritability. First, there is the potential for inflation of 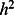 estimates based on closely related individuals such as MZ/DZ twins. It is well known that epistatic interactions can inflate heritability estimates in studies of related individuals [13]. Recent work from Zuk et al [10] has examined this in detail. Other factors that could also lead to inflated estimates of 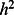 using closely related pairs of individuals include dominance and shared environment. Second, there is a tradeoff between inflation and sampling variance when estimating 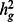. The recent variance component approach described by Yang et. al results in inflated estimates of 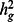 in the presence of related individuals [12], [14], [15], [16], [17]. However, removing related individuals reduces the sample size, resulting in a larger standard error around the estimate [18], [19]. Both of these issues can adversely affect estimates of missing heritability.

Here, we analyze the heritability of 23 complex phenotypes in an Icelandic cohort of 38,167 individuals, leveraging both a population-wide genealogical database and genotype data from over 300,000 SNPs that have been long-range phased across and between chromosomes (i.e. where not only the phase, but also the parental origin of alleles has been determined) [20]. Importantly, we develop an approach that allows 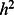 to be estimated on the basis of both closely and distantly related pairs of individuals. We find, for all of the quantitative phenotypes, that our estimates of 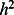 are smaller than those from the literature that were based on MZ/DZ twins [21]. Our results indicate that previous estimates were inflated by the impact of epistasis or shared environment.

We further introduce a new variance components method that provides simultaneous estimates of 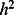 and 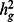. This method has two principal advantages. First, by adequately taking account of both closely and distantly related pairs of individuals, it minimizes the standard error of the estimates, whilst avoiding the upward bias that can result from calculations based on closely related pairs. Second, it produces both estimates of heritability for the same population sample, ensuring that 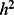 and 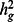 are directly comparable.

For most of the 23 phenotypes examined here, our results show that 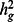 accounts for more than half of 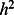. As GWAS have not identified many SNPs with large effect sizes (i.e. 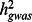 is small), and 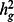 is greater than 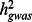 by a considerable margin, it follows that there must be many associated sequence variants that remain to be discovered, i.e. these phenotypes are highly polygenic. Currently, only common variants are well captured by the genotyping arrays used in most GWAS studies. As the difference between 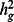 and 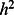 is likely due to common and rare variants not captured by the genotyping array [12], it may be assumed that a fair number of association signals remain to be identified through more comprehensive approaches, such as whole genome-sequencing. However, our estimates of 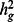 show that GWAS genotyping arrays capture a greater proportion of 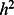 than indicated by previous twin-based estimates of 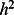.

## Results

### Overview of methods

Below, we provide an overview of the approaches we used to estimate various components of heritability. The details of these approaches are provided in the Methods section.

We used a linear mixed model approach to estimate components of heritability. In this approach, each phenotype is modeled using a multivariate normal distribution. Each of the components of heritability that we estimated corresponds to a different model of the phenotypic covariance.

Narrow-sense heritability (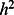) estimates from variance component models rely on covariance matrices specifying the genome-wide genetic relatedness of individuals in the data set. An estimate of 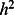 can be obtained by using an identity-by-descent (IBD) based covariance matrix, which is trivial to obtain from long-range phased genotype data (see below).

The fine-scale estimates of IBD used here rely on long-range phasing data that are not available in most data sets. An estimate of 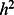 can also be obtained by using an identity-by-state with threshold (IBS>t) based covariance matrix with all values below a threshold t set to 0, i.e. focusing on closely related individuals. An alternative is to use the full IBS based covariance matrix to obtain an estimate of the heritability explained by genotyped SNPs (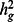), however, this requires removing related individuals [12]. If related individuals are included, the resulting estimate is neither an estimate of 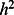 nor an estimate of 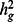.

Previous approaches to estimating the heritability explained by genotyped SNPs (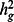) required filtering related individuals, thereby increasing the standard error of the estimates. However, joint estimates of 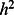 and 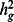 can be obtained using two covariance matrices based on IBS>t and IBS. The first component provides an estimate of 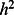, and the second provides an estimate of 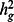. This approach removes the need to filter related individuals. Alternately, joint estimates of 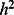 and 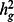 can be obtained using two covariance matrices based on IBD and IBS, where here IBD replaces IBS>t to estimate 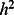.

Broad-sense heritability (H^2^) is the sum of additive, dominant, and epistatic components of heritability. The additive, dominant, environmental (ADE) model can be used to obtain joint estimates of dominance and additive components of heritability, using two covariance matrices based on IBD2 (two copies shared IBD) and IBD [22].

Below, we investigate all of these modeling approaches. Table S1 contains definitions of all parameters quantifying components of heritability that are used in the text.

### Estimates of narrow-sense heritability (h^2^)

Ideally, estimates of narrow-sense heritability of a particular phenotype would be based on a genetic relationship matrix constructed from causal variants, representing the true genetic contribution to the phenotype [23]. However, as this set of variants is typically not known for most phenotypes, a proxy must be used for the pair-wise genetic covariance of individuals at the causal variants. Traditionally, this proxy has been derived from genealogical information, representing, for each pair of individuals in a sample, the expected fraction of their genomes that is identical-by-descent (IBD) – i.e. identical as a result of being inherited from a recent common ancestor [23]. The availability of dense genome-wide data from microarray SNP genotyping platforms has made it possible to directly estimate the fraction of the genome shared IBD between each pair of individuals (**K**_IBD_). However, fine-scale estimation of **K**_IBD_ in population samples is dependent on information about the chromosomal phase of alleles, which requires long-range phasing of the data [17], [24], [25]. Previous studies reporting 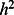 estimates in close relatives based on **K**_IBD_ [26], [27], [28] had very high standard errors based on their study design and sample size. Recent work has examined IBD-based heritability estimates from distantly related individuals [29]. Ours is the first study to provide fine-scale IBD-based estimates of 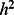 based on pairs of individuals at a range of relationship from siblings to distant relatives. We refer to these estimates as 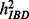.

IBD-based estimates of 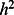 for the 11 quantitative traits (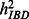) are shown in Table 1. For these and subsequent estimates of h^2^, age, sex, and geographic region were included as covariates to prevent confounding. These estimates range from 0.099 for recombination rate to 0.691 for height. The only quantitative trait yielding an estimate not significantly different from 0 was sex-ratio of offspring. For each of the eight quantitative phenotypes with published estimates of 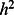, our estimates were smaller than the mean published estimate. For example, our estimate of 0.69 for height was lower than previous estimates of 0.80 [30], but was consistent with previous estimates in being lower for females (0.724 s.e. 0.019) than males (0.780 s.e. 0.029) [31].

**Table 1:** Narrow-sense heritability estimated from IBD (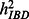) and thresholding IBS (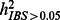) for 11 quantitative traits.

Quantitative trait	N^a^	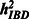	s.e.	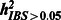	s.e.	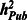
Body Mass Index (kg/m^2^)	20000	0.422	0.018	0.433	0.018	0.4–0.6 [6]
Cholesterol High Density Lipoprotein	19977	0.446	0.017	0.457	0.018	0.5 [6]
Cholesterol Low_Density Lipoprotein	4547	0.196	0.062	0.198	0.063	0.376 [42]
Height (cm)	20000	0.691	0.016	0.704	0.016	0.8 [6]
Menarche Age (years)	15150	0.443	0.022	0.454	0.022	0.4–0.7 [43]
Menopause Age (years)	5540	0.400	0.047	0.409	0.048	0.4–0.6 [44]
Monocyte White Blood Cell	9651	0.343	0.032	0.351	0.032	0.378 [42]
Waist-Hip Ratio	5538	0.181	0.037	0.187	0.038	0.3–0.6 [45]
Sex Ratio of offspring	15000	0.026	0.017	0.021	0.018	-
Total Children	15000	0.103	0.017	0.111	0.018	-
Recombination Rate	10259	0.099	0.023	0.110	0.030	-

a N is the number of individuals used in the analysis of each phenotype. 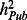 are previously published estimates of heritability from different populations.

Previous studies, based on either genealogical or direct estimates of IBD sharing, have been limited to closely related individuals (first-cousins or closer), and may therefore be upwardly biased due to the impact of shared environment, dominance, or epistatic interactions [10]. On average, our estimates of 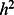 were lower than those from previous studies by a ratio of 0.75 (s.e. 0.067), most likely because the latter were inflated by one of the three aforementioned factors. We return to this point below, performing a pedigree-based analysis to assess the impact of these factors. Dichotomous phenotypes in this study were ascertained to increase the number of available cases, leading inflation in 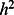. A discussion of this inflation and the resulting estimates are presented in Text S1 and Table S2.

In most cases, researchers do not have access to long-range phased genotypes with which to estimate h^2^. One suggested solution to this problem is the use of **K**_IBS_, the genome-wide proportion of alleles shared identical-by-state (IBS) at all genotyped loci, as a substitute for **K**_IBD_ [32], when estimating 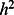 on the basis of closely-related pairs of individuals (it is assumed that **K**_IBS_ provides a poor estimate of **K**_IBD_ for distantly related pairs of individuals). Taking advantage of long-range phase based estimates of **K**_IBD_, we sought to evaluate the use of **K**_IBS_ for the estimation of h^2^. For this purpose, we computed **K**_IBS_ as defined in [12] and found that it produced downwardly biased estimates of 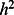 for both simulated and real data sets that included many pairs of distantly related individuals (see Methods). As noted by Vattikuti et al [19], this bias is due to the fact that, when used to estimate h^2^, the **K**_IBS_ matrix captures information from two distinct sources, depending on the degree of relationship between pairs of individuals. For large values of IBS it estimates genetic covariance over all SNPs in the genome. For low values of IBS it estimates genetic covariance over just those SNPs on the genotyping platform 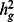 (see next section). Thus, the resulting heritability estimates from **K**_IBS_ therefore tend to lie between the true value, 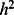, and the typically lower value of 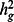.

To avoid this bias, we implemented a different approach, retaining all individuals for the calculation of h^2^, but setting values of **K**_IBS_ less than or equal to a threshold t (**K**_IBS>t_) to 0, for t = 0.00, 0.025 and 0.05. This threshold defines the separation between closely and distantly related individuals. We evaluated this approach using both simulations and real data sets and observed a significant downward bias of narrow-sense heritability estimated from tresholded IBS (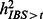) at t = 0. For example, when t = 0 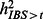 for height is 0.58, while when t = 0.05 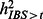 = 0.70 (similar results were obtained for the other phenotypes). We observed no bias at t = 0.025 or t = 0.05 (see Methods and Table S3). To err on the side of caution, we present 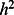 values for t = 0.05 (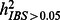) in Table 1 and Table S2, for the quantitative and dichotomous traits, respectively. The difference between narrow-sense heritability estimated from IBD (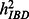 ) and 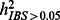 was less than 0.015 for all traits and not statistically significant for any of them. The correlation between the two estimators was 0.9998 and 0.9999 for the quantitative and dichotomous traits, respectively. Furthermore, in our extensive simulations over real data, the difference between the estimators was always less than 0.02 and not statistically significant (see Methods and Tables S3, S4, S5). These results indicate that when phase information is not available **K** *_IBS_* can provide unbiased and precise estimates of h^2^, by means of 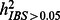, in data consisting of a mixture of closely and distantly related pairs of individuals. The choice of threshold t is a function of the relatedness structure of the individuals in the study as well as the properties of the population they are drawn from (see Discussion).

### Joint estimation of 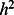 and 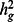 for quantitative phenotypes

Recently, Yang et al [12] developed a method for estimating 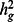, the fraction of narrow sense heritability explained by genotyped SNPs (and SNPs in LD with genotyped SNPs). The interest in 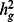 derives from the fact that it is the upper bound on the heritability that can be described from GWAS (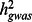) conducted on the same genotyping platform used to estimate 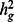. The Yang et al. method is based on a variance component model with a genetic relationship matrix **K** *_IBS_* estimated from the genotyped SNPs. To prevent inflation, the method requires that all pairs of individuals have **K** *_IBS_*<0.025 [12]. In studies where the Yang et al. [12] approach has been applied [18], [19], the removal of related individuals resulted in a significant decrease in sample size and a concomitant increase in the standard error of the heritability estimates (e.g. a standard error of 19% in one study [18]). Filtering such that **K** *_IBS_*<0.025 for all individuals in our data leaves less than 3000 individuals, which is not adequate to estimate 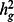 with low standard error(for example, resulting in a standard error for 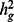 of 10.0% for height).

To enable unbiased calculation of 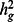 in data sets that contain a both closely and distantly related pairs of individuals, we have devised an alternative approach based on a model with two variance components (see Methods). The first variance component, **K** *_IBS_* has a parameter 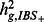 and is an estimate of 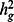, the genetic variance due genotyped SNPs. The second variance component **K** *_IBS_*, has a parameter 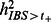 and is an estimate of 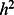, the total narrow-sense heritability (the subscript+is used for both parameters to denote that they are estimated simultaneously). Although we have access to fine-scale estimates of **K** *_IBD_*, based on long-range phased genotype data, we demonstrate the application of this approach using **K** *_IBS_*_>t_, because fine-scale **K** *_IBD_* estimates are typically not available to most researchers. We note that in the empirical results and in simulation, the use of **K** *_IBD_* and **K** *_IBS_* in the model produced results that were similar to those obtained using **K** *_IBS_*_>t_ and **K** *_IBS_* (see Methods). Extensive testing of this model was performed to demonstrate that it estimates the appropriate quantities (see Methods), and estimates of 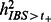 closely match those of narrow-sense heritability estimated from tresholded IBS and IBD (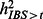 and 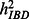), both in our data and in simulations.

Table 2 shows heritability results for quantitative traits using the joint model where heritability estimated from thresholding IBS (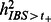) and heritability explained by genotyped SNPs (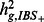) are fit jointly. We examined the nine quantitative traits where h^2^>0. Our results are concordant with the previous estimates of 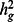 for height, high-density lipoprotein (HDL), WHR, and BMI [6], [19]. For most of the traits, 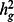 accounts for more than half of 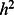, with a maximum of 0.75 for waist-to-hip ratio (WHR), and a minimum of 0.33 for age at menopause. For each trait, we tested for deviation from a 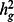/h^2^ ratio of 0.53 (the average across all the traits) and found that only height, with a value of 0.58 was significantly different (p-value<0.0017, see Text S1). However, as our estimates of 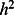 were smaller than previous estimates, the fraction 0.53 (s.e. 0.042) of variance explained by genotyped SNPs based on our estimates of heritability was larger than the fraction 0.40 (s.e. 0.037) based on published estimates [6].

**Table 2:** Heritability estimated from thresholding IBS (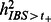) and heritability explained by genotyped SNPs (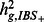).

Phenotype	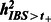	s.e.	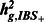	s.e.	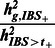	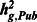
Body Mass Index	0.424	0.018	0.229	0.017	0.540	0.16(0.03) [46]
Cholesterol High Density Lipoprotein	0.450	0.017	0.239	0.017	0.531	0.12(0.05) [19]
Cholesterol Low_Density Lipoprotein	0.199	0.063	0.103	0.065	0.518	-
Height	0.687	0.016	0.399	0.017	0.581	0.42(0.03) [46]
Menarche Age	0.451	0.022	0.225	0.022	0.499	-
Menopause Age	0.409	0.048	0.136	0.053	0.333	-
Monocyte White Blood Cell	0.343	0.032	0.198	0.032	0.577	-
Waist Hip Ratio	0.188	0.037	0.140	0.055	0.745	0.13(0.05) [19]
Total Children	0.102	0.028	0.043	0.023	0.422	-

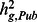
 are previously reported estimates of 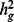 with standard errors given in ()'s.

### Joint estimation of narrow-sense and heritability explained by genotyped SNPs for dichotomous phenotypes

For dichotomous phenotypes, ascertainment in samples with closely related pairs of individuals induces an upward bias in narrow-sense heritability jointly estimated from IBS above a threshold (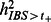) when converting to the liability scale (Table S6 and Text S1). Thus, our 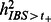 estimates should be viewed as an upper bound. However, it is possible to account for case-control ascertainment amongst distantly related pairs when converting heritability explained by genotyped SNPs jointly estimated from IBS below a threshold (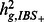) from the observed to the liability scale [33]. This correction is not possible when affected relatives are included in the study. For example, a study that ascertains affected sib pairs will have severely inflated heritability estimates, and the case-control ascertainment correction does not address this type of bias (see Text S1). Table 3 shows 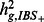 estimates on the liability scale, derived from a model with two variance components K*_IBS>t_* and K*_IBS_*, for 11 dichotomous traits. Estimates of 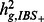 primarily capture the heritability derived from distantly related pairs of individuals. Results on the observed scale are given in Table S7. The inflated narrow-sense heritability estimates of the dichotomous phenotypes leads to a lower ratio of heritability explained by genotyped SNPs (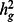) to 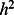.

**Table 3:** Narrow-sense heritability explained by genotyped SNPs (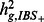) for dichotomous phenotypes on the liability scale.

Phenotype	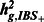	s.e.	Prevalence	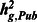
Alcohol Dependence	0.235	0.030	0.07	
Asthma	0.264	0.067	0.13	
Autoimmune Systemic RA SLE SSc AS	0.200	0.048	0.02	
Autoimmune Tcell mediated	0.192	0.033	0.05	
Breast Cancer	0.117	0.051	0.12	
Coronary Artery Disease	0.146	0.017	0.06	0.39(0.06)
Hypertension in Pregnancy	0.083	0.043	0.03	
Osteoarthritis	0.126	0.026	0.1	
Prostate Cancer	0.204	0.056	0.09	
Rheumatoid Arthritis**	0.261	0.061	0.01	0.63(0.06)0.32(0.07)*
Type 2 Diabetes	0.254	0.041	0.08	0.44(0.06)

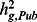
 are previously reported estimates of 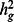 with standard errors given in ()'s.

* RA estimate without the MHC region.

** RA in our study contained a mixture CCP positive and negative cases, while the previously published worked is based on CCP positive cases only [34].

Our results are lower than previous estimates of the heritability explained by genotyped SNPs (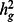) for rheumatoid arthritis (RA), 2 diabetes (T2D), and coronary artery disease (CAD) [34]. The differences between our estimates and previous estimates could be due to the use of population controls in our study rather than non-affected controls, differences in disease prevalence between populations, differences in the genotyping platform used, differences in ascertainment strategies such as age matching in previous work, or actual differences in the heritability of the phenotype across populations. If a small number of common variants were responsible for a large fraction of the phenotypic variation, they would have been identified by previous GWAS. However, since most of the loci identified through GWAS have a small effect, our results suggest a highly polygenic model of disease for the dichotomous phenotypes, as in the case of the quantitative traits. This is consistent with previous studies [6].

### Estimation of heritability explained by shared environment, dominance, and epistasis

Shared environment, dominance effects and epistasis (i.e. non-additive interaction between variants) can upwardly bias estimates of 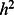 in data sets that contain closely related pairs of individuals [10]. Phenotypic covariance in siblings can be strongly affected by dominance effects, and as siblings have correlated phenotypes due to shared environment, these two factors are strongly confounded [11]. In addition, Zuk et al [10] showed that epistatic interactions also lead to inflated estimates of 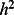 and is an additional confounding factor. Inflation due to any of these factors affects interpretation of the relationship between 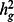 and 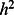 and is likely to result in overestimates of missing heritability when 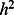 is estimated using closely related pairs of individuals. We adopted two approaches to test for evidence of 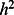 inflation and to determine the extent to which it could be accounted for by shared environment, epistasis, or dominance effects (referred to collectively hereafter as dominance-like effects).

First, we estimated additive and dominance-like effects simultaneously under an ADE (additive, dominant, and environmental) model with variance components **K** *_IBD_* and **K** *_IBD_*_2_, where the latter represents the fraction of the genome with both chromosomes shared IBD for each pair of individuals [26]. A likelihood ratio test of the ADE model against the single variance component model of **K** *_IBD_* was performed (see Methods) [35], producing two heritability estimates 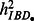 the narrow sense heritability, and 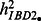 the heritability due to dominance-like effects (the subscript “
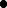
”denotes that these two estimates are generated simultaneously from the same model). The sum of these two estimates is the broad sense heritability *H* ^2^. The only class of relationship with significant IBD2 is siblings who share an expected ¼ of their genome IBD2. This analysis will therefore focus overwhelmingly on the difference between siblings and other classes of relationship. Siblings are also subject to epistatic interactions and shared environment and so this analysis will be influenced by all three factors (shared environment, dominance, and epistasis). We note that this analysis will not detect shared environment effects that decay exactly in proportion to genome-wide IBD.

We initially examined a subset of 11 quantitative and dichotomous traits, viewed as likely candidates for environmental effects, in a subset of 15,000 genotyped individuals using the ADE framework. The results for these phenotypes are shown in Table 4, with heritability estimates for dichotomous traits given on the observed scale. Six phenotypes exhibited 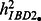 that was significantly greater than zero, with an average value of 0.37, indicating the impact of dominance-like effects. Hypertension in pregnancy, T2D, CAD and osteoarthritis showed the strongest effects (see Figure S1). While these results give clear evidence of inflation of h^2^, they do not distinguish between the possible sources of inflation. The fact that the narrow sense heritability estimate 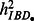 decreases when fit jointly with 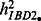 demonstrates that the IBD based estimated conducted over relatives is susceptible to inflation.

**Table 4:** Joint estimates of heritability from two copy (dominant) IBD (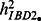) and singly copy (additive) IBD (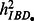).

Phenotype	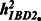	s.e.	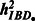	s.e.	N^a^	p-value
Body Mass Index	0.090	0.069	0.381	0.023	15000	0.18
Coronary Artery Disease	0.387	0.078	0.164	0.023	6661 CA 11774 CO	3.36E-04
Cholesterol High Density Lipoprotein	0.141	0.066	0.423	0.023	15000	0.03
Cholesterol Low Density Lipoprotein	0.257	0.071	0.202	0.023	13121	2.81E-04
Osteoarthritis	0.279	0.075	0.181	0.022	2319 CA 11666 CO	3.66E-05
Type 2 Diabetes	0.363	0.072	0.301	0.022		2.86E-08
Total number of children	0.073	0.068	0.095	0.019	15000	0.27
Total number of children (Mothers)	0.180	0.066	0.145	0.020	15000	4.19E-03
Breast Cancer	0.154	0.081	0.128	0.022	2214 CA 11687 CO	0.05
Prostate Cancer	0.296	0.082	0.144	0.027	1792 CA 8328 CO	9.01E-03
Hypertension in Pregnancy	0.826	0.074	0.072	0.021	419 CA 10085 CO	3.33E-16

For dichotomous phenotypes these estimates are inlineed on the observed scale. ^a^N is the number of individuals used in the analysis of each phenotype (CA = cases; CO = control). The p-values are from the likelihood ratio test of the ADE model against the **K** *_IBD_* model, with values less than 0.05 implying the presence of environmental, dominance, and/or epistatic interaction effects (i.e. 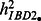
>0).

**Figure 1:**
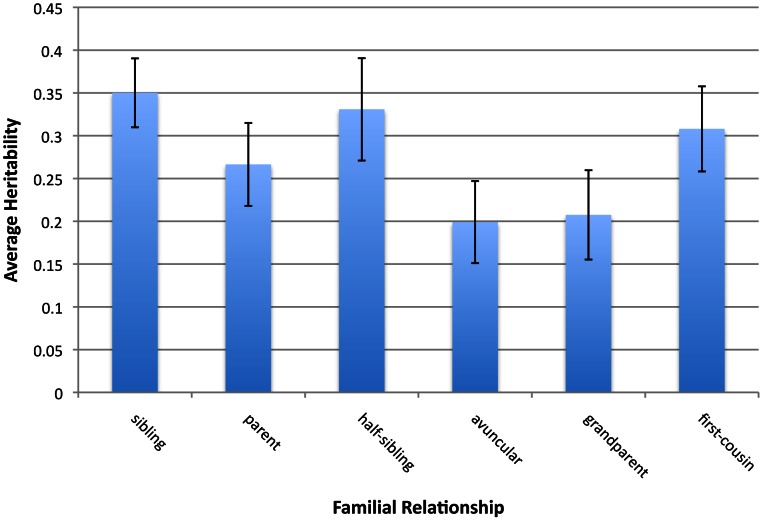
Average heritability estimates and 95% confidence intervals of 17 phenotypes for six classes of relationship. The differences in heritability estimate between classes of relationship are consistent with a shared-environment only effect on phenotypic correlation, but not with a dominance only or epistasis only effect on phenotypic correlation.

In order to address this issue, we performed a pedigree-based analysis, making use of genealogical information [36] to assess the effect of shared environment against those of dominance and epistasis. For each class of family relationship, we estimated 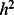 for each phenotype by dividing the phenotypic correlation by the genealogical expectation for the fraction of genome shared IBD (see Methods). Errors in the genealogical database are believed to be small but may bias the estimates, especially for the more distant relatives [36]. Each source of inflation is expected to generate a distinct pattern of heritability estimates across the relationship classes. We extended our analyses to 17 of the original 23 phenotypes where, for each class of familial relationship, there were at least 100 pairs of individuals for continuous phenotypes and at least 50 cases and 50 controls for dichotomous phenotypes (the full set of phenotypes is given in Text S1). Figure 1 and Table S8 reveal a gradient of average 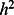 estimates across the 17 phenotypes, ranging from 0.35 for sibs to 0.2 for avuncular pairs, that is inconsistent with dominance or epistasis being the sole source of 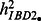, either individually or in combination. The large standard errors prevent more detailed conclusions about the relative contributions of environment and epistasis. It is possible that maternal and paternal half siblings have different environmental sharing, but due to their low numbers we analyzed them together.

If the heritability estimate of two copy IBD when fit jointly with IBD (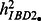) were due to dominance, one would expect all classes of relationship to exhibit the same value of 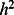 with the exception of siblings, but this is not what is observed. (Siblings are the only class with significant **K**_IBD2_ due to dominance and would have larger estimates of 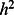 than any other class of relationship.) If 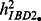
were due to epistasis, one would expect a monotonic relationship between 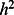 and **K**_IBD_ [10], such that all classes sharing the same fraction of the genome IBD (such as half-siblings and grandparent-grandchild pairs) yield the same estimate of h^2^. Again, this is not what is observed. Finally, if 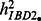 were due to shared environment, one would expect that relationship classes that entail considerable shared environment through cohabitation (sibs, half-sibs and parent-offspring) would have greater values of 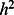 than relationship classes with the same fraction of IBD but no cohabitation. Indeed, this is what is observed.

Our results show that 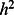 estimates for half-siblings are greater than those of grandparent-grandchild pairs by 0.125 on average. This suggests that dominance or epistasis are not the sole sources of 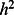 inflation in data sets containing many closely related pairs of individuals. Figure 1 does not imply that first cousins share more similar environments than parent and offspring or siblings. The first-cousin phenotypic correlation is multiplied by a factor of eight to estimate heritability, while the parent-offspring correlation is only multiplied by two. Thus, first-cousins could share substantially less similar environments (nearly four times less similar), and still have a higher estimate of heritability as a result.

Two additional results from Table S8 deserve further attention. First, the greater 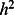 estimates obtained for parent-offspring than for avuncular pairs are consistent with shared environment. Second, the greater 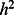 estimates obtained for first cousins than for grand-relatives and avuncular pairs (both have a twofold greater value of *K_IBD_*), suggests that first cousins have shared environmental factors stronger than either of these other classes of relationship. Although we condition on age a strong non-linear effect of age on phenotype, or a cohort effect, could produce this elevated correlation amongst first cousin phenotypes.

## Discussion

We have made use of long-range phased genotype data and genealogical information from an Icelandic cohort to shed light on the problem of missing heritability, and the relative contributions of common and rare sequence variants and environmental factors to complex human phenotypes.

First, we examined IBD based estimation of narrow-sense heritability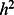 in data containing both closely and distantly related individuals. Our estimates were lower than previously published estimates, which were primarily based on closely related pairs of individuals. This suggests that previous estimates may be upwardly biased and that the fraction of variance described by known associations detected by GWAS of common variants is greater than previously thought. We also showed that estimates of 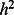 based on thresholding IBS (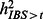) were nearly identical to those based on IBD estimates derived from long-range phased genotype data. Thus, we demonstrate that it is possible to estimate 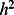 using either IBD or IBS>t in cases where long-range phased genotypes are not available.

Second, we developed a new method to estimate 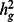 (the heritability explained by genotyped SNPs), based on both closely and distantly related pairs of individuals, which has the additional advantage of providing a joint and directly comparable estimate of h^2^. The estimated value of 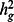 is an upper bound on the amount of variation that can be described by SNPs on a given genotyping platform and is driven almost entirely by common variation. Previously, it was necessary to prune related individuals from data sets prior to calculation, substantially increasing the standard error around the estimate [19], [37]. In the case of height in our data set, the pruning approach resulted in a standard error for 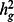 of 10%. In comparison, our method produced one of only 1.7%.

Finally, we investigated the impact of shared environment, dominance, and epistasis on estimates of 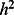 in data sets that include pairs of closely related individuals. We found that 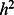 estimates for several phenotypes, including type 2 diabetes and coronary artery disease, were significantly inflated due to such dominance-like effects. By examining patterns of correlation across multiple classes of relationship, we have demonstrated that the effects of shared environment outweigh those of epistasis and dominance. However, our results indicate that shared environment may be the major contributor to inflated values of 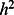 obtained with data sets that include closely related pairs of individuals. They also suggest that this inflation, as opposed to consistently lower heritabilities in Iceland, is the major source of difference between our estimates those of previously studied populations. MZ/DZ twin estimates of 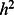 assume that the two classes of siblings share the same relevant environmental exposures. If this is true, then inflation from such studies may be due instead to epistasis.

A standard way to quantify the contribution of environmental effects is to fit an ACE model [13]. However, a complexity with this approach is that it is unclear which relative classes should be modeled as sharing a common environment. For example, do parent/child pairs have the same environmental sharing as siblings? We believe this merits further investigation, although it is outside the scope of our current work.

Interestingly, our estimate of the heritability of height (0.69) is lower than previous estimates (0.8) [30] based on studies of twins, siblings, parent-offspring, half-siblings, and first-cousins. Visscher [26], [27] previously used estimates of IBD amongst siblings instead of the expected value of 0.5 to estimate the heritability of height. The standard error of his estimate (mean 0.8, standard error 0.1) was too large for this estimate to be statistically different from either 0.69 or 0.8. We note that this estimate would be inflated in the presence of epistasis since the study focuses on siblings. Zuk et al [10] proposed that heritability estimates of closely related individuals maybe inflated in the presence of epistasis, but an epistasis-only explanation would require a deflation in estimates moving from closely related individuals (siblings) to more distant relationships (first-cousins), which is not observed. One possible explanation is inflation in previous estimates of height due to a combination of epitasis and shared environment across multiple levels of relationship (e.g. siblings to first-cousins). These sources of inflation would be reduced when more distantly related individuals are available as is uniquely the case in this study (see Text S1). Heritability may vary between segments of the population, such as males and females. In this work we chose not to subdivide the population into segments, but instead make our estimates in the entire population. The covariates we used (age, sex, geographic region) will account for mean differences but not differences in variance or heritability between these groups.

We conclude that, for quantitative traits, more than half of 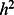 is explained by genotyped SNPs. Because of our smaller estimates of h^2^, this fraction is larger than previous estimates [6], [19], [34]. It is encouraging to learn that more can be discovered using the common variants on microarray SNP genotyping platforms used in GWAS than some recent pessimistic reports have concluded [38], [39]. One potential reason for the differences between 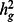 and 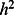 is that rare variation accounts for a significant fraction of the total narrow-sense heritability. If this is the case, then genome-wide sequencing studies offer a potential route to capturing the remaining heritability.

## Methods

### Ethics statement

This research was approved by the Data Protection Commission of Iceland and the National Bioethics Committee of Iceland. The appropriate informed consent was obtained for all sample donors.

### deCODE data set

We analyzed 38,167 individuals from the deCODE data set genotyped on the Illumina 300K chip. Owing to the sensitive nature of genotype data, access to these data can only be granted at the headquarters of deCODE Genetics in Iceland. The details of the genotyping, quality control, IBD estimation and genealogy are described elsewhere [20], [28]. . The 11 quantitative phenotypes examined in this study are body mass index (BMI), high density lipoprotein cholesterol (HDL), low density lipoprotein cholesterol (LDL), height, age at menarche, age at menopause, monocyte white blood cell count, waist hip ratio (WHR), sex ratio, number of offspring, and recombination rate (see Table 1). The 11 dichotomous phenotypes are alcohol dependence, asthma, autoimmune Systemic RA+SLE+SSc+AS (rheumatoid arthritis, systemic lupus erythematosus, systemic sclerosis, ankylosing spondylitis), T-cell mediated autoimmune disease, breast cancer (BC), coronary artery disease (CAD), hypertension in pregnancy, osteoarthritis, prostate cancer (PC), rheumatoid arthritis (CCP positive and negative) (RA), type 2 diabetes (T2D), and left handedness (see Table S2). Dichotomous phenotypes are diagnosed by physicians with the exception of left-handedness, which is measured by self-report. Continuous phenotypes are measured by health professionals, medical laboratories, and an extended genealogy [36]. The exceptions are age at menopause and menarche, which are measured by self-report.

The deCODE Genetics genealogy database, containing all contemporary Icelanders and most of their ancestors going back to the year 1650, was used to determine the genealogical relationships between individuals [36].

A description of the phenotypes is given in Text S1. Estimation of IBD2 is analogous to estimation of IBD, but only includes instances where individuals are IBD on both chromosomes.

### Statistical methods

We used a linear mixed model approach to estimate the components of heritability in our data sets. In this approach, each phenotype Y is modeled from a multivariate normal distribution 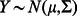 where 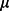 is the mean phenotype. Each of the components of heritability described correspond to a different model for the phenotypic covariance matrix 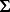.

For a normalized phenotype with mean 0 and variance 1, to estimate 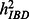, we set 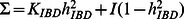 and find the estimate of 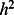 that maximized the restricted maximum likelihood (REML) of Y being generated from 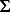 using the GCTA software [40]. We applied the same method to estimate 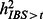 and 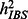 using the kinship matrices **K**_IBS>t_ and **K**_IBS_ respectively. To jointly estimate 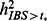
+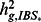 we fit Y with 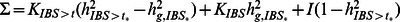. We applied the same method to estimate 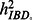
+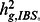 using kinship matrices **K**_IBD_ and **K**_IBS_. The intuition for this approach is that the large elements of genetic covariance matrix **K**_IBS_ are good estimates of the pair-wise IBD of individuals. Indeed, this is why 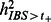 is a good estimate of 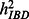. However, the small elements of **K**_IBS_ only provide information about SNPs in LD with those on the genotyping platform. This is why the **K**_IBS_ applied to unrelated individuals in the approach of Yang et al. estimates 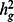. By breaking **K**_IBS_ into two components, one provides estimates of the phenotypic variance explained by SNPs on the genotyping platform, and the other provides an estimate of the remaining phenotypic variance. The total narrow-sense heritability is the sum of the parameters for **K**_IBS>t_ and **K**_IBS_, and the heritability explained by genotyped SNPs is the parameter for **K**_IBS_.

To estimate 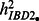 and 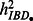 we fit Y with 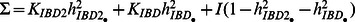. The parameter for **K**_IBD2_ is a combination of shared environment, dominance, and epistatic effects. The parameter for **K**_IBD_ is the narrow-sense heritability. Under the assumption of no shared environment or epistatic effects, the sum of these estimates is the broad-sense heritability. In all cases we adjusted for age, gender, and region of Iceland as fixed effects. We use REML including a constant vector instead of ML to prevent bias in heritability estimation [35]. We estimated the prevalence of each dichotomous phenotype as the fraction of cases in the entire cohort.

The **K**_IBS_ matrix is estimated as defined in [12]. Entry j,k is
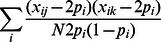
(1)where xij is the genotype (0,1,2), N is the number of SNPs, and pi is the minor allele frequency of SNP i in the study. Entry j,j is an estimate of the inbreeding coefficient 
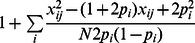
. **K**_IBS>t_ is the **K**_IBS_ matrix with all entries less than t set to zero, with the exception of the diagonal, which is not changed.

Entry (i,j) of the **K**_IBD_ matrix is the fraction of the genome shared IBD between individuals i and j. Pair-wise IBD estimates were estimated as described in [28]

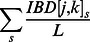
(2)where L is the length of the genome and IBD [j,k]at nucleotide s is 1 if individuals j,k are IBD at position s and 0 otherwise. Entry (j,j) is 1+ the fraction of the genome shared IBD between the maternal and paternal copies. The IBS matrices are all adjusted to have mean zero (Equation 1) and the IBD matrices are not (Equation 2). Entry (i,j) of the **K**_IBD2_ matrix is defined similarly, but pair-wise IBD2 estimates (both chromosomes IBD) are used in place of pair-wise IBD estimates. That is, a pair of individuals is IBD2 at a particular SNP if they are IBD on both haplotypes. Entry, (j,j) is set to 1.0. We note that none of the kinship matrices defined here, and hence none of the resulting heritability estimates rely on the genealogy, but are based on direct estimates from the genetic data.

### Simulations with simulated genotypes

We performed a set of experiments over simulated genotype data in order verify that our estimates of 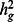 and our 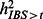 estimates of 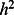 were estimating the correct heritabilities. We generated simulated observed genotypes of individuals as random draws from a binomial distribution with minor allele frequencies p_1_,p_2_,…,p_N_ where the number of SNPs N = 5,000 and pi drawn uniformly between 0.05 and 0.5. We then repeated this process to create unobserved genotypes for each individual. The observed genotypes represent those on a genotyping platform and the unobserved represent those not in LD with the genotyped SNPs.

To simulate a pair individuals with x% of their genome shared IBD, we copied x% of the SNPs of the first individual's haplotypes onto the corresponding SNPs of the second individual in the pair. We normalized the genotypes to have mean 0 and variance 1 and set the effect size for each SNP to be the square root of 0.5/10000. The phenotype for each individual is the sum over all SNPs of the product of the normalized genotype and the effect size plus noise drawn from a random distribution with mean 0 and variance 0.5. This gives a phenotype with mean 0, variance 1, a of heritability 50%, of which 50% is due to observed genotypes and 50% is due to unobserved genotypes.

We then constructed relationship matrices K using the observed genotypes. For **K**_IBD_, we assumed that we had access to the true value of IBD for each pair of individuals. We constructed data sets of 1,400 individuals with several different types of relationship structure, created 1,000 replicates of each data set, and estimated narrow-sense heritability 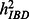 using the IBD matrix **K**_IBD_, 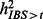 using the IBS matrix **K**_IBS>t_, and 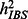 using the complete IBS matrix **K**_IBS_. We estimated the joint estimates 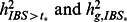 of 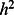 and 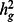 using the 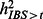 approximation for IBD, and compared this to the joint estimates 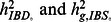, which use IBD directly.

The results shown in Table S3 demonstrate that **K**_IBD_ and **K**_IBS>t_ give good estimates of 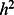 when there are closely related individuals in the data set, but have a high variance when the relationships are more distant. **K**_IBS_ is not a good estimator of either 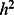 or 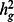 when there are related individuals in the data set, since it lies in between 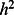 and 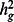. Both **K**_IBD_+**K**_IBS_ and **K**_IBS>t_+**K**_IBS_ provide joint estimates of 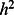
+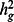 without the need to remove related individuals. When one of every pair of individuals with **K**_IBS_>0.025 is removed from the data set, the estimate of 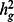 has the same mean as when estimated simultaneously with **K**_IBS>t_, but the variance is significantly higher.

When the related individuals are closely related (e.g. K = 0.5), **K**_IBS>t_ is a good estimator of KIBD and the mean heritability estimate is the true 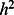. However, while pair-wise IBS is a good estimate of the pair-wise IBD, the variance of the IBS estimate is distributed according to the observed genotypes. The heritability estimate from **K**_IBS_ is therefore biased towards the heritability explained by genotyped SNPs. This is why 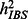 always lies in between 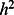 and 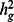 and using **K**_IBS_ to estimate narrow-sense heritability without thresholding can lead to biased heritability estimates.

When the relatedness of individuals in the data set is moderate (e.g. K = 0.125), the joint model **K**_IBS>t_+**K**_IBS_ does not provide a good estimate of since **K**_IBS>t_ will be influenced by genotyped variants. However, 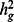 is still unbiased and the variance of 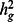 is lower than it would have been if related individuals were removed. Therefore, we have a means of including distantly related individuals when estimating 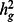. The value of **K**_IBS>t_ as an estimate for the true heritability depends on the relatedness structure of the data set. In data sets with families, such as the cohort examined here, or the FHS data set [19], it is possible to estimate both 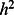 and 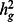. In data sets with moderate relatedness, the robustness of the thresholding approach should be examined via simulation.

### Simulations with real genotypes

We performed a similar set of experiments to those described above, but this time used real genotype data with simulated phenotypes in order to verify that issues due to LD, IBD estimation, population structure, or other similar confounders did not affect our results. We selected 8000 individuals randomly from the complete data set and generated two sub-phenotypes for each individual. We generated two sets of causal variants C_1_ and C_2_ by selecting a causal variant every 500 SNPs along the even chromosomes for C_1_ and repeating the process along the odd chromosomes for C_2_. We chose effect sizes *α_1_* and *α_2_* for C_1_ and C_2_ respectively and set the sub-phenotypes for an individual by summing the product of their genotypes (0,1,2) times the corresponding effect sizes. We then added random noise *ε_1_* and *ε_2_* to each sub-phenotype and summed together the two sub-phenotypes to generate a final phenotype for each individual. The even chromosomes correspond to the observed genotypes in a GWAS and the odd chromosomes correspond to SNPs not in LD with the genotyped SNPs. For any choice of *α_1_* and *α_2_*, and variances of *ε_1_* and *ε_2_* we can compute the heritability of the phenotype due to all SNPs 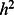 and the heritability explained by genotyped SNPs 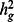.

We recomputed the relationship matrices K using only the even chromosomes. For each simulated phenotype we estimated heritability using **K**_IBD_, **K**_IBS_, **K**_IBS>t_, and **K**_IBS>t_+**K**_IBS_. The results are shown in Tables S5, S7 and as was the case for the simulated synthetic data sets above, the heritability estimates are within one standard deviation of the true value in all cases with the exception of **K**_IBS_, which always lies between 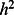 and 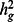. We confirm the bias of using **K**_IBS_ by examining the estimate of 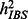 in several real phenotypes (Table S9). We observe that it always lies between 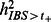 and 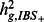.

### Heritability estimation of classes of related individuals

For a given class of relatives, (e.g. siblings), for each phenotype we computed the correlation between the phenotype across all pairs of that class. The heritability estimate was then generated by dividing the correlation by the fraction of the genome expected to be shared IBD (e.g. 0.5 for siblings). It is not possible to place standard errors on the heritability estimate of the phenotypes due to the complex relatedness structure of the individuals in each class. One pair of siblings for example, might be the grandfather and granduncle of another pair of siblings. However, it is possible to compute an empirical mean and standard deviation across traits.

To compare the classes of relatives we computed the empirical mean and standard deviation of the differences of the heritability estimates across traits. The standard error of the difference is the standard deviation estimate divided by the square root of the number of phenotypes (17 in this case). We applied a Wald test to determine the p-value [41].

To determine the significance of the combined effects of shared environment, dominance, and epistatic interaction we constructed a one degree of freedom likelihood ratio test. We computed the likelihood of the ADE model fit with covariance matrices **K**_IBD2_+**K**_IBD_ and the likelihood of the narrow-sense heritability estimated from **K**_IBD_.

### Upward biases in narrow-sense heritability estimates

The heritability estimates in Table 1 and Table S2 may be upwardly biased due to shared environment since closely related individuals will have correlated phenotyped due to shared genetics as well as shared environment. The heritability estimates on the liability scale in Table S2 may be additionally upwardly biased due to sample ascertainment. As affected individuals in this analysis were non-randomly ascertained with respect to disease status and family relationship (for example, the probability of ascertaining two affected sibs is greater than that of ascertaining two affected first-cousins.), all 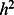 estimates based on related pairs of individuals will tend to be inflated. We converted heritability estimates from the observed to liability scale (see Table S3) using the prevalences shown in Table S2 [33], noting that these estimates represent an upper bound. To our knowledge, no method exists to adjust 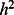 estimates for this type of ascertainment.

## Supporting Information

Figure S1 Average heritability estimates of type 2 diabetes, coronary artery disease, and hypertension in pregnancy for six classes of relationship. The differences in heritability estimate between classes of relationship are consistent with a shared-environment only effect on phenotypic correlation, but not with a dominance only or epistasis only effect on phenotypic correlation.
(PDF)

Table S1 Definition of subscripted parameters.
(DOCX)

Table S2 Narrow-sense heritability (*h^2^*) for 12 dichotomous traits on the liability scale.
(DOCX)

Table S3 Heritability estimates over simulated data for a range of relatedness structures. Model refers to the relatedness structure of the data. 0.5 represents 700 pairs of individuals with 50% of their genome IBD. 0.5,0.25 represents 350 pairs with 50% IBD and 350 pairs with 2.5% IBD. 0.125,0.025 represents 350 pairs with 12.5% IBD and 350 pairs with 2.5% IBD. We used a threshold *t* = 0.05.
(DOCX)

Table S4 Heritability estimates from data simulated over even and odd chromosomes of 8,000 individuals from the decode cohort.
(DOCX)

Table S5 Heritability estimates from data simulated over even and odd chromosomes of 8,000 individuals from the decode cohort.
(DOCX)

Table S6Narrow-sense heritability estimated from thresholding IBS (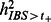). Dichotomous narrow-sense heritability estimates are inflated due to ascertainment and shared environment.(DOCX)

Table S7Narrow-sense heritability (*h^2^*) for 12 dichotomous traits on the observed scale. 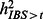 is estimates from the single variance component model using the thresholded IBS matrix.(DOCX)

Table S8Differences in heritability estimates between pairs of classes of relationships. If there is no effect of shared environment, dominance, or epistatic interaction then 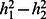 should equal 0. *sib* represents sib-pairs, avuncular represents uncle/aunt-niece/nephew, and grandparent represents grandparent-grandchild. We note that the significance values are more a measure of power than of model.(DOCX)

Table S9IBS based estimates (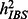) for a subset of phenotypes demonstrates that 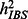 are biased upward from 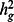 and downward from 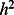.(DOCX)

Text S1 Using extended genealogy to estimate components of heritability for 23 quantitative and dichotomous traits.
(DOCX)
