# Supplementary figures and images for: Using Extended Genealogy to Estimate Components of Heritability for 23 Quantitative and Dichotomous Traits
Source: PLoS Genet. 2013 May 30;9(5):e1003520. doi: 10.1371/journal.pgen.1003520 (PMC3667752; doi:10.1371/journal.pgen.1003520)

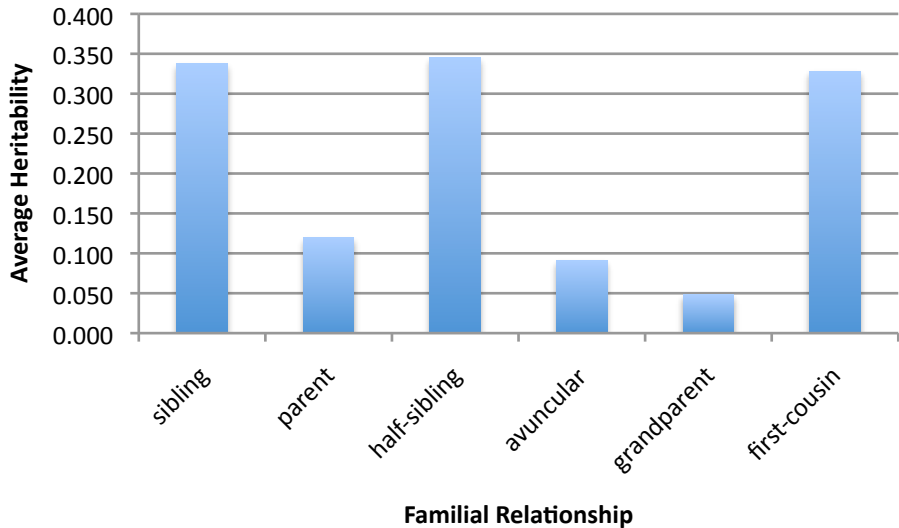

Supplement: Figure S1 — Average heritability estimates of type 2 diabetes, coronary artery disease, and hypertension in pregnancy for six classes of relationship. The differences in heritability estimate between classes of relationship are consistent with a shared-environment only effect on phenotypic correlation, but not with a dominance only or epistasis only effect on phenotypic correlation. (PDF) [file pgen.1003520.s001.pdf]
