# Supplementary material for: Using Extended Genealogy to Estimate Components of Heritability for 23 Quantitative and Dichotomous Traits
Source: PLoS Genet. 2013 May 30;9(5):e1003520. doi: 10.1371/journal.pgen.1003520 (PMC3667752; doi:10.1371/journal.pgen.1003520)
Supplement: Table S1 — Definition of subscripted parameters. (DOCX) [file pgen.1003520.s002.docx]

Table S1: Definition of subscripted parameters

| Symbol | Definition |
| --- | --- |
| $h^{2}$ | Narrow-sense heritability |
| $h_{g}^{2}$ | Heritability explained by genotyped SNPs |
| $h_{gwas}^{2}$ | Heritability explained by significantly associated SNPs from previous GWAS |
| 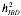 | Estimate of narrow-sense heritability using fine-scale estimate of IBD |
| $h_{IBS}^{2}$ | Biased heritability estimated using IBS over both related and unrelated individuals |
| $h_{IBS>t}^{2}$ | Estimate of narrow sense heritability using IBS values greater than a threshold *t*. |
| $h_{IBS>t_{+}}^{2}$ | Estimate of narrow-sense heritability from the joint variance component model containing IBS > *t* and IBS. |
| $h_{g,IBS_{+}}^{2}$ | Estimate of heritability of genotyped SNPs from the joint variance component model containing IBS > *t* and IBS. |
| 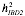 | Estimate of combination of dominance, shared environment, and epistasis computed from two copy IBD sharing. |
| 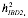 | Same as 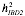 when fit in the joint model with IBD2 and IBD. |
| 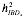 | Same as 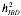 when fit in the joint model with IBD2 and IBD. |
