# Supplementary material for: Using Extended Genealogy to Estimate Components of Heritability for 23 Quantitative and Dichotomous Traits
Source: PLoS Genet. 2013 May 30;9(5):e1003520. doi: 10.1371/journal.pgen.1003520 (PMC3667752; doi:10.1371/journal.pgen.1003520)
Supplement: Table S2 — Narrow-sense heritability (h2) for 12 dichotomous traits on the liability scale. (DOCX) [file pgen.1003520.s003.docx]

Table S2. Narrow-sense heritability (*h^2^*) for 12 dichotomous traits on the liability scale.

| **Dichotomous traits** | **Cases** | **Controls** | **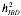** | **s.e.** | **$h_{IBS>t}^{2}$** | **s.e.** | Prevalence | $h_{Pub}^{2}$ |
| --- | --- | --- | --- | --- | --- | --- | --- | --- |
| Alcohol Dependence | 2909 | 17091 | 0.641 | 0.038 | 0.656 | 0.038 | 0.07 | 0.30-0.36^4^ |
| Asthma | 1473 | 18527 | 1.852 | 0.080 | 1.902 | 0.080 | 0.13 | 0.53-0.91^5^ |
| Autoimmune Systemic RA SLE SSc AS | 1074 | 18926 | 0.958 | 0.058 | 0.984 | 0.058 | 0.02 | - |
| Autoimmune Tcell mediated | 2292 | 17708 | 0.941 | 0.038 | 0.968 | 0.038 | 0.05 | - |
| Breast Cancer | 1917 | 12574 | 0.388 | 0.056 | 0.403 | 0.056 | 0.12 | 0.3^6^ |
| Coronary Artery Disease | 6322 | 13678 | 0.273 | 0.018 | 0.279 | 0.018 | 0.06 | 0.3-0.6^7^ |
| Hypertension in Pregnancy | 1366 | 18634 | 0.767 | 0.055 | 0.788 | 0.058 | 0.03 | 0.22^8^ |
| Osteoarthritis | 4096 | 15904 | 0.656 | 0.028 | 0.673 | 0.028 | 0.1 | 0.39-0.65^9^ |
| Prostate Cancer | 1766 | 6151 | 0.465 | 0.056 | 0.479 | 0.057 | 0.09 | 0.42-0.57^10^ |
| Rheumatoid Arthritis | 748 | 19252 | 1.203 | 0.069 | 1.230 | 0.069 | 0.01 | 0.53-0.68*^7^(0.13 MHC) |
| Type 2 Diabetes | 2165 | 17835 | 0.932 | 0.043 | 0.960 | 0.046 | 0.08 | 0.3-0.6^6^ |
| Left Handedness | 366 | 4545 | 0.004 | 0.145 | 0.011 | 0.153 | 0.1 | - |

$h_{Pub}^{2}$ are previously published estimates of heritability from different populations. *Dichotomous narrow-sense heritability estimates are inflated due to ascertainment and shared environment.
