# Supplementary material for: Using Extended Genealogy to Estimate Components of Heritability for 23 Quantitative and Dichotomous Traits
Source: PLoS Genet. 2013 May 30;9(5):e1003520. doi: 10.1371/journal.pgen.1003520 (PMC3667752; doi:10.1371/journal.pgen.1003520)
Supplement: Table S3 — Heritability estimates over simulated data for a range of relatedness structures. Model refers to the relatedness structure of the data. 0.5 represents 700 pairs of individuals with 50% of their genome IBD. 0.5,0.25 represents 350 pairs with 50% IBD and 350 pairs with 2.5% IBD. 0.125,0.025 represents 350 pairs with 12.5% IBD and 350 pairs with 2.5% IBD. We used a threshold t = 0.05. (DOCX) [file pgen.1003520.s004.docx]

Table S3:Heritability estimates over simulated data for a range of relatedness structures. Model refers to the relatedness structure of the data. 0.5 represents 700 pairs of individuals with 50% of their genome IBD. 0.5,0.25 represents 350 pairs with 50% IBD and 350 pairs with 2.5% IBD. 0.125,0.025 represents 350 pairs with 12.5% IBD and 350 pairs with 2.5% IBD. We used a threshold *t*=0.05.

| Model | 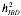 | var | 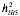 | var | 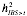 | var |
| --- | --- | --- | --- | --- | --- | --- |
| 0.5 | 0.510 | 0.029 | 0.432 | 0.014 | 0.503 | 0.027 |
| 0.5,0.25 | 0.504 | 0.034 | 0.385 | 0.011 | 0.492 | 0.031 |
| 0.125,0.025 | 0.546 | 0.307 | 0.268 | 0.009 | 0.348 | 0.140 |

| Model |  | var |  | var | $h_{IBS>t_{*}}^{2}$ | var | $h_{g,IBS_{*}}^{2}$ | var |
| --- | --- | --- | --- | --- | --- | --- | --- | --- |
| 0.5 | 0.510 | 0.044 | 0.242 | 0.012 | 0.510 | 0.042 | 0.244 | 0.012 |
| 0.5,0.025 | 0.504 | 0.048 | 0.251 | 0.011 | 0.504 | 0.045 | 0.253 | 0.012 |
| 0.125,0.025 | 0.490 | 0.346 | 0.245 | 0.010 | 0.357 | 0.163 | 0.246 | 0.011 |
