# Supplementary material for: Using Extended Genealogy to Estimate Components of Heritability for 23 Quantitative and Dichotomous Traits
Source: PLoS Genet. 2013 May 30;9(5):e1003520. doi: 10.1371/journal.pgen.1003520 (PMC3667752; doi:10.1371/journal.pgen.1003520)
Supplement: Table S4 — Heritability estimates from data simulated over even and odd chromosomes of 8,000 individuals from the decode cohort. (DOCX) [file pgen.1003520.s005.docx]

Table S4:Heritability estimates from data simulated over even and odd chromosomes of 8,000 individuals from the decode cohort.

| $h_{IBD}^{2}$ | s.e | $h_{IBS>t}^{2}$ | s.e. | $h_{IBS}^{2}$ | s.e. | h^2^ |
| --- | --- | --- | --- | --- | --- | --- |
| 0.76 | 0.03 | 0.78 | 0.03 | 0.35 | 0.03 | 0.80 |
| 0.78 | 0.04 | 0.79 | 0.04 | 0.31 | 0.03 | 0.80 |
| 0.83 | 0.03 | 0.84 | 0.03 | 0.43 | 0.03 | 0.80 |
| 0.75 | 0.04 | 0.77 | 0.04 | 0.41 | 0.03 | 0.80 |
| 0.78 | 0.03 | 0.79 | 0.04 | 0.51 | 0.02 | 0.80 |
| 0.75 | 0.04 | 0.76 | 0.04 | 0.52 | 0.02 | 0.80 |
| 0.80 | 0.04 | 0.82 | 0.04 | 0.58 | 0.02 | 0.80 |
| 0.78 | 0.04 | 0.79 | 0.04 | 0.55 | 0.02 | 0.80 |
| 0.72 | 0.04 | 0.74 | 0.04 | 0.69 | 0.02 | 0.80 |
| 0.80 | 0.03 | 0.82 | 0.04 | 0.68 | 0.02 | 0.80 |
