# Supplementary material for: Using Extended Genealogy to Estimate Components of Heritability for 23 Quantitative and Dichotomous Traits
Source: PLoS Genet. 2013 May 30;9(5):e1003520. doi: 10.1371/journal.pgen.1003520 (PMC3667752; doi:10.1371/journal.pgen.1003520)
Supplement: Table S5 — Heritability estimates from data simulated over even and odd chromosomes of 8,000 individuals from the decode cohort. (DOCX) [file pgen.1003520.s006.docx]

Table S5: Heritability estimates from data simulated over even and odd chromosomes of 8,000 individuals from the decode cohort.

| $h_{IBS>t_{*}}^{2}$ | s.e. | 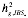 | s.e. | $h_{g}^{2}$ | $h^{2}$ |
| --- | --- | --- | --- | --- | --- |
| 0.84 | 0.04 | 0.20 | 0.03 | 0.20 | 0.80 |
| 0.75 | 0.04 | 0.19 | 0.03 | 0.20 | 0.80 |
| 0.80 | 0.04 | 0.29 | 0.03 | 0.30 | 0.80 |
| 0.76 | 0.04 | 0.28 | 0.03 | 0.30 | 0.80 |
| 0.82 | 0.04 | 0.41 | 0.03 | 0.40 | 0.80 |
| 0.77 | 0.04 | 0.42 | 0.03 | 0.40 | 0.80 |
| 0.76 | 0.04 | 0.51 | 0.03 | 0.50 | 0.80 |
| 0.80 | 0.04 | 0.48 | 0.03 | 0.50 | 0.80 |
| 0.77 | 0.04 | 0.64 | 0.03 | 0.60 | 0.80 |
| 0.77 | 0.04 | 0.61 | 0.03 | 0.60 | 0.80 |
