# Supplementary material for: Using Extended Genealogy to Estimate Components of Heritability for 23 Quantitative and Dichotomous Traits
Source: PLoS Genet. 2013 May 30;9(5):e1003520. doi: 10.1371/journal.pgen.1003520 (PMC3667752; doi:10.1371/journal.pgen.1003520)
Supplement: Table S6 — Narrow-sense heritability estimated from thresholding IBS (). Dichotomous narrow-sense heritability estimates are inflated due to ascertainment and shared environment. (DOCX) [file pgen.1003520.s007.docx]

Table S6: Narrow-sense heritability estimated from thresholding IBS ($h_{IBS>t_{+}}^{2}$). Dichotomous narrow-sense heritability estimates are inflated due to ascertainment and shared environment.

| Phenotype | 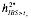 | s.e. |
| --- | --- | --- |
| Alcohol Dependence | 0.653 | 0.038 |
| Asthma | 1.898 | 0.080 |
| Autoimmune Systemic RA SLE SSc AS | 0.984 | 0.058 |
| Autoimmune Tcell mediated | 0.964 | 0.038 |
| Breast Cancer | 0.401 | 0.056 |
| Coronary Artery Disease | 0.274 | 0.018 |
| Hypertension in Pregnancy | 0.785 | 0.058 |
| Osteoarthritis | 0.670 | 0.028 |
| Prostate Cancer | 0.479 | 0.057 |
| Rheumatoid Arthritis^**^ | 1.230 | 0.069 |
| Type 2 Diabetes | 0.947 | 0.046 |
