# Supplementary material for: Using Extended Genealogy to Estimate Components of Heritability for 23 Quantitative and Dichotomous Traits
Source: PLoS Genet. 2013 May 30;9(5):e1003520. doi: 10.1371/journal.pgen.1003520 (PMC3667752; doi:10.1371/journal.pgen.1003520)
Supplement: Table S7 — Narrow-sense heritability (h2) for 12 dichotomous traits on the observed scale. is estimates from the single variance component model using the thresholded IBS matrix. (DOCX) [file pgen.1003520.s008.docx]

Table S7: Narrow-sense heritability (*h^2^*) for 12 dichotomous traits on the observed scale. **$h_{IBS>t}^{2}$** is estimates from the single variance component model using the thresholded IBS matrix.

| **Dichotomous traits** | **Cases** | **Controls** | **$h_{IBD}^{2}$** | **s.e.** | **$h_{IBS>t}^{2}$** | **s.e.** |
| --- | --- | --- | --- | --- | --- | --- |
| Alcohol Dependence | 2909 | 17091 | 0.339 | 0.020 | 0.347 | 0.020 |
| Asthma | 1473 | 18527 | 0.442 | 0.019 | 0.454 | 0.019 |
| Autoimmune Systemic RA SLE SSc AS | 1074 | 18926 | 0.297 | 0.018 | 0.305 | 0.018 |
| Autoimmune Tcell mediated | 2292 | 17708 | 0.450 | 0.018 | 0.463 | 0.018 |
| Breast Cancer | 1917 | 12574 | 0.160 | 0.023 | 0.166 | 0.023 |
| Coronary Artery Disease | 6322 | 13678 | 0.263 | 0.017 | 0.269 | 0.017 |
| Hypertension in Pregnancy | 1366 | 18634 | 0.267 | 0.019 | 0.274 | 0.020 |
| Osteoarthritis | 4096 | 15904 | 0.406 | 0.017 | 0.417 | 0.017 |
| Prostate Cancer | 1766 | 6151 | 0.317 | 0.038 | 0.326 | 0.039 |
| Rheumatoid Arthritis | 748 | 19252 | 0.314 | 0.018 | 0.321 | 0.018 |
| Type 2 Diabetes | 2165 | 17835 | 0.367 | 0.017 | 0.378 | 0.018 |
| Left Handedness | 366 | 4545 | 0.001 | 0.038 | 0.003 | 0.040 |
