# Supplementary material for: Using Extended Genealogy to Estimate Components of Heritability for 23 Quantitative and Dichotomous Traits
Source: PLoS Genet. 2013 May 30;9(5):e1003520. doi: 10.1371/journal.pgen.1003520 (PMC3667752; doi:10.1371/journal.pgen.1003520)
Supplement: Table S8 — Differences in heritability estimates between pairs of classes of relationships. If there is no effect of shared environment, dominance, or epistatic interaction then should equal 0. sib represents sib-pairs, avuncular represents uncle/aunt-niece/nephew, and grandparent represents grandparent-grandchild. We note that the significance values are more a measure of power than of model. (DOCX) [file pgen.1003520.s009.docx]

Table S8: Differences in heritability estimates between pairs of classes of relationships. If there is no effect of shared environment, dominance, or epistatic interaction then
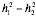
 should equal 0. *sib* represents sib-pairs, avuncular represents uncle/aunt-niece/nephew, and grandparent represents grandparent-grandchild. We note that the significance values are more a measure of power than of model.

| Relation1 | Relation 2 | $h_{1}^{2}-h_{2}^{2}$ | s.e. | p-value |
| --- | --- | --- | --- | --- |
| sib | half-sib | 0.019 | 0.056 | 0.73 |
| sib | first-cousin | 0.042 | 0.028 | 0.13 |
| sib | grandparent | 0.143 | 0.039 | 2.93E-04 |
| sib | parent | 0.084 | 0.026 | 1.42E-03 |
| sib | avuncular | 0.151 | 0.026 | 3.72E-09 |
| half-sib | first-cousin | 0.023 | 0.060 | 0.70 |
| half-sib | grandparent | 0.123 | 0.056 | 0.03 |
| half-sib | parent | 0.064 | 0.063 | 0.31 |
| half-sib | avuncular | 0.132 | 0.059 | 0.03 |
| first-cousin | grandparent | 0.100 | 0.044 | 0.02 |
| first-cousin | parent | 0.042 | 0.044 | 0.35 |
| first-cousin | avuncular | 0.109 | 0.038 | 4.23E-03 |
| grandparent | parent | -0.059 | 0.030 | 0.05 |
| grandparent | avuncular | 0.008 | 0.029 | 0.77 |
| parent | avuncular | 0.067 | 0.017 | 7.61E-05 |
