# Supplementary material for: Using Extended Genealogy to Estimate Components of Heritability for 23 Quantitative and Dichotomous Traits
Source: PLoS Genet. 2013 May 30;9(5):e1003520. doi: 10.1371/journal.pgen.1003520 (PMC3667752; doi:10.1371/journal.pgen.1003520)
Supplement: Table S9 — IBS based estimates () for a subset of phenotypes demonstrates that are biased upward from and downward from . (DOCX) [file pgen.1003520.s010.docx]

Table S9. IBS based estimates (
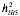
) for a subset of phenotypes demonstrates that
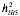
 are biased upward from
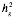
 and downward from
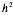
.

| Phenotype | $h_{IBS>t_{+}}^{2}$ | s.e. | 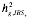 | s.e. | 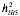 | s.e. |
| --- | --- | --- | --- | --- | --- | --- |
| Cholesterol High Density Lipoprotein | 0.450 | 0.017 | 0.239 | 0.017 | 0.361 | 0.013 |
| Cholesterol Low_Density Lipoprotein | 0.199 | 0.063 | 0.103 | 0.065 | 0.162 | 0.047 |
| Height | 0.687 | 0.016 | 0.399 | 0.017 | 0.574 | 0.012 |
| Rheumatoid Arthritis | 0.321 | 0.018 | 0.068 | 0.016 | 0.197 | 0.013 |
