# Supplementary material for: Using Extended Genealogy to Estimate Components of Heritability for 23 Quantitative and Dichotomous Traits
Source: PLoS Genet. 2013 May 30;9(5):e1003520. doi: 10.1371/journal.pgen.1003520 (PMC3667752; doi:10.1371/journal.pgen.1003520)
Supplement: Text S1 — Using extended genealogy to estimate components of heritability for 23 quantitative and dichotomous traits. (DOCX) [file pgen.1003520.s011.docx]

**Supplementary Text S1**

*Estimates of narrow-sense heritability* (*h*^2^) *for dichotomous phenotypes*

Table S2 shows $h_{IBD}^{2}$ on the liability scale for the 12 dichotomous traits. Here, only handedness provided an estimate that was not significantly different from 0. Heritabilities of dichotomous traits are typically estimated on the liability scale, because heritability on the observed scale varies as a function of prevalence^1^. For example, two diseases with prevalences 0.5 and 0.01, completely driven by genetics (liability-scale $h^{2}$ = 1.0) will have observed-scale heritabilities of 0.637 and 0.072, respectively. Due to the over-ascertainment of cases in our study, the heritabilities for dichotomous phenotypes are inflated, and therefore represent an upper bound of the true heritability.

*Fraction of heritability explained by* $h_{g}^{2}$

The average ratio of $h_{g}^{2}$/*h*^2^  was 0.53 across 10 quantitative phenotypes in the study. Sex ratio was removed because its heritability estimate could not be differentiated from 0. For each phenotype we tested whether the observed ratio$h_{g}^{2}$/*h*^2^ was consistent with a model in which the true ratio was 0.53. We generated 100,000 values of $\hat{h}_{g}^{2}$ drawn from a normal distribution with mean equal to 0.53 times the observed heritability $h^{2}$ and standard error equal to the observed standard error of $h_{g}^{2}$. In the case of BMI this was a mean of 0.223 and a standard error of 0.017. For each $\hat{h}_{g}^{2}$ we computed a ratio by dividing by the observed heritability $h^{2}$ (0.424 for BMI). We then estimated two p-values, the fraction of the 100,000 tests that were less than the observed ratio, and the fraction that were greater than the observed ratio. Only height had a statistically significant result with less than 0.17% of generated ratios greater than the observed ratio of 0.581.

We also tested whether the distribution of ratios $h_{g}^{2}$/*h*^2^ across all 11 quantitative phenotypes was consistent with a model in which the true ratio was 0.53 for all phenotypes. We simulated 100,000 sets of $\hat{h}_{g}^{2}$ of 11 phenotypes with means equal to 0.53 times the observed heritabilities $h^{2}$ and standard errors equal to the observed standard errors of $h_{g}^{2}$. For each set we computed the standard error of the 11 $\hat{h}_{g}^{2}$draws. We found the probability that the observed standard error of the 11 observed $h_{g}^{2}$ was within the generated distribution with p-value = 0.287. Thus, based on the distribution of $h_{g}^{2}$/*h*^2^ ratios, we cannot rule out a model in which the true ratio is 0.53 for all phenotypes. We chose to use simulation based test as opposed to a Wald because we did not want to make assumptions of the distribution of $h_{g}^{2}$/*h*^2^.

*Correcting for case-control ascertainment and ascertainment of affected relatives*

Heritability for case-control traits is computed on the observed scale. Case-control ascertainment can alter the observed scale heritability estimate and so liability scale estimates are often reported so that they may be compared between studies of differing ascertainment strategies. Lee et al^1^ recently demonstrated how to convert observed scale estimates to liability scale estimates when using a variance component model over unrelated individuals. We employ their approach for our estimates of heritability explained by genotyped chips ($h_{g}^{2}$ ), which relies only on unrelated individuals.

However, the Lee et al approach does not correct for ascertainment of affected relatives. For example, a study that ascertains affected sib pairs will have severely inflated estimates, and the Lee et al. correction does not address this type of bias. Our estimates of narrow-sense heritability ($h^{2}$ ), rely on the use of related individuals. The ascertainment strategy for inclusion of individuals in this study used both relationship class and case-control status. This prevents the application of the Lee et al approach since the probability of inclusion is not just a function of disease status. The liability scale estimates are therefore inflated and are reported as upper bounds.

*17 Phenotypes used in “Distinguishing between shared environment, dominance and epistasis”*

We sub-selected 17 phenotypes from the complete set of 23 phenotypes that had at least 100 individuals in each relationship class examined for continuous phenotypes or 50 cases and 50 controls for dichotomous phenotypes. The phenotypes that remained after filtering were: alcohol dependence, asthma, autoimmune RA SLE SSc AS, autoimmune T Cell mediated, BMI, BC, HDL, LDL, Height, hypertension in pregnancy, age at menarche, white blood cell count, osteoarthritis, PC, RA, T2D, and WHR.

*Heritability of Height*

In this work we estimated the heritability of height to be 0.69. The heritability of height has previously been consistently estimated at approximately 0.8 across a number of studies including mono versus di-zygotic twins, sibling studies, parent-child studies, half-sibling studies, and studies of first cousins^2^. Interestingly, in a meta-analysis across studies including all of these relationship classes, the slope of the regression of phenotypic-correlation versus expected genetic relationship led to a y-intercept of 0.107 instead of 0^2^. This is driven in part by an increased heritability of height at the level of first-cousins, and was previously explained by a combination of assortative mating and social homogamy^2^. Another possibility is a combination of epistatic interactions and shared environmental factors.

A shared environmental factor, such as diet, that extends to the level of first-cousins will cause more inflation at distant relationships. Under the assumption of a purely additive model of phenotype, siblings have heritability equal to two times their phenotypic correlation whereas first-cousins have heritability equal to eight times their phenotypic correlation. Thus, if the increase in phenotypic correlation due to a shared environmental factor does not reduce less than four fold between siblings and first-cousins it will cause an inflation in heritability estimates at the level of first-cousins relative to siblings. Monozygotic twins and siblings produce roughly the same heritability estimate, which is not expected under a shared environment only model. However, monozygotic twins will have significantly more inflation in the presence of epistatic interaction than siblings, and similarly for siblings relative to first-cousins. The combination of both of these factors can by combined to produce the meta-analysis regression observed in [^2^].

Heritability estimates from distant relationships will not be inflated due to either shared environment or epistatic interactions, except in the case of regional environmental effects. This study includes many thousands of pairs of distant relatives and has a lower heritability estimate of 0.69. Another possibility for our lower estimate of the heritability of height, which we consider less likely, is that the true heritability of height is smaller in Iceland than other European populations.

*Description of Phenotypes*

The 11 quantitative phenotypes examined in this study are body mass index (BMI), high density lipoprotein cholesterol (HDL), low density lipoprotein cholesterol (LDL), height, age at menarche, age at menopause, monocyte white blood cell count, waist hip ratio (WHR), sex ratio, number of offspring, and recombination rate (see Table 1). The 11 dichotomous phenotypes are alcohol dependence, asthma, autoimmune Systemic RA+SLE+SSc+AS (rheumatoid arthritis, systemic lupus erythematosus, systemic sclerosis, [ankylosing spondylitis](http://en.wikipedia.org/wiki/Ankylosing_Spondylitis)), T-cell mediated autoimmune disease, breast cancer (BC), coronary artery disease (CAD), hypertension in pregnancy, osteoarthritis, prostate cancer (PC), rheumatoid arthritis (CCP positive and negative) (RA), type 2 diabetes (T2D), and left handedness (see Table S2). Dichotomous phenotypes are diagnosed by physicians with the exception of left-handedness, which is measured by self-report. Continuous phenotypes are measured by health professionals, medical laboratories, and an extended genealogy^3^. The exceptions are age at menopause and menarche, which are measured by self-report.

**Bibliography**

1 Lee, S. H., Wray, N. R., Goddard, M. E. & Visscher, P. M. Estimating Missing Heritability for Disease from Genome-wide Association Studies. *Am J Hum Genet* **88**, 294-305, doi:S0002-9297(11)00020-6 [pii]

10.1016/j.ajhg.2011.02.002 (2011).

2 Visscher, P. M., McEvoy, B. & Yang, J. From Galton to GWAS: quantitative genetics of human height. *Genet Res (Camb)* **92**, 371-379, doi:S0016672310000571 [pii]

10.1017/S0016672310000571 (2010).

3 Helgason, A., Palsson, S., Gudbjartsson, D. F., Kristjansson, T. & Stefansson, K. An association between the kinship and fertility of human couples. *Science* **319**, 813-816, doi:319/5864/813 [pii]

10.1126/science.1150232 (2008).

4 Walters, G. D. The heritability of alcohol abuse and dependence: a meta-analysis of behavior genetic research. *Am J Drug Alcohol Abuse* **28**, 557-584 (2002).

5 Thomsen, S. F., van der Sluis, S., Kyvik, K. O., Skytthe, A. & Backer, V. Estimates of asthma heritability in a large twin sample. *Clin Exp Allergy* **40**, 1054-1061, doi:CEA3525 [pii]

10.1111/j.1365-2222.2010.03525.x (2010).

6 Visscher, P. M., Brown, M. A., McCarthy, M. I. & Yang, J. Five years of GWAS discovery. *Am J Hum Genet* **90**, 7-24, doi:S0002-9297(11)00533-7 [pii]

10.1016/j.ajhg.2011.11.029 (2012).

7 Stahl, E. A. *et al.* Bayesian inference analyses of the polygenic architecture of rheumatoid arthritis. *Nat Genet*, doi:ng.2232 [pii]

10.1038/ng.2232 (2012).

8 Thornton, J. G. & Macdonald, A. M. Twin mothers, pregnancy hypertension and pre-eclampsia. *Br J Obstet Gynaecol* **106**, 570-575 (1999).

9 Kirk, K. M. *et al.* The validity and heritability of self-report osteoarthritis in an Australian older twin sample. *Twin Res* **5**, 98-106, doi:10.1375/1369052022965 (2002).

10 Lichtenstein, P. *et al.* Environmental and heritable factors in the causation of cancer--analyses of cohorts of twins from Sweden, Denmark, and Finland. *N Engl J Med* **343**, 78-85 (2000).
